# Elevated troponin levels as a predictor of mortality in patients with acute stroke: a systematic review and meta-analysis

**DOI:** 10.3389/fneur.2024.1351925

**Published:** 2024-03-25

**Authors:** Annu Gulia, Manyata Srivastava, Pradeep Kumar

**Affiliations:** Clinical Research Unit, All India Institute of Medical Sciences, New Delhi, India

**Keywords:** troponin, mortality, acute stroke, ischemic stroke, intracerebral hemorrhage, subarachnoid hemorrhage

## Abstract

**Background and Aim:**

The prognostic potential of cardiac troponin (cTn) in acute stroke patients has been a subject of ongoing debate. Our objective was to provide a comprehensive evidence for predicting mortality in acute stroke patients by using the elevated troponin levels.

**Methods:**

We conducted an extensive literature search, including PubMed, EMbase, and Trip Databases, covering studies published up to September 30, 2023. We computed risk ratios (RR) with 95% confidence intervals (CIs), performed sensitivity analysis, and conducted trial sequential analysis (TSA).

**Results:**

In total, 53 studies were analyzed, with 37 focusing on acute ischemic stroke (AIS), 11 on subarachnoid hemorrhage (SAH), and 7 on Intracerebral hemorrhage (ICH). Elevated cTn levels were significantly showed a higher predictive risk for In-hospital mortality in both AIS (RR=3.80, 95% CI; 2.82 to 5.12) as well as SAH (RR=2.23, 95% CI; 1.64 to 3.02). However, no significant predictive risk between elevated cTn levels and in-hospital mortality for ICH patients (RR=1.13, 95% CI: 0.46 to 2.79). A similar pattern was observed for elevated cTn levels, indicating an increased risk of last follow-up mortality for AIS (RR=2.41, 95% CI: 1.98 to 2.93) and SAH (RR=3.08, 95% CI: 2.25 to 4.21).

**Conclusion:**

Elevated troponin levels can serve as a promising predictive marker for both in-hospital and last follow-up mortality in AIS and SAH patients but not in ICH patients. Further prospective studies are needed to validate our findings along with exploring the preventive management of mortality in acute stroke settings.

## Introduction

Acute stroke represents a critical medical condition with substantial implications for patient outcomes and healthcare systems (1). In this context, the timely and accurate identification of prognostic markers capable of predicting stroke-associated mortality emerges as a paramount imperative (2). Such markers not only facilitate risk assessment but also guide precise and targeted clinical interventions, ultimately influencing patient outcomes and optimizing healthcare resource allocation (3). Cardiac Troponin (cTn), widely recognized as a cardinal biomarker in cardiology, has recently attracted attention for its potential role as an indicator of mortality risk in patients afflicted by acute stroke (4).

In the short term, the immediate post-stroke period, in-hospital mortality rates for acute stroke patients can exhibit alarming elevations, with figures occasionally reaching a staggering 50% (5). The major driving force behind such acute fatalities often lies in cardiac-related complications. These complications manifest through the release of cardiac troponin-T (cTnT), cardiac troponin-I (cTnI), High-sensitive cardiac Troponin I (hs-cTnI) and High-sensitive cardiac Troponin T (hs-cTnT) proteins into the circulatory system. Importantly, during the acute phase of ischemic stroke (IS), a marked elevation in serum levels of cTnT or cTnI is frequently observed, establishing a robust link between cardiac injury and the stroke itself (6). The precise and timely prognosis of this cardiac involvement not only impacts therapeutic strategies but also plays a pivotal role in the monitoring and management of patient outcomes, fundamentally shaping the quality of healthcare delivery (7).

However, despite the potential of cardiac troponin as a prognostic marker, the scientific literature presents contrasting findings regarding its association with the risk of all-cause mortality in patients afflicted with acute stroke (8). Moreover, the precision of risk estimates demonstrates considerable variability across individual research studies. Troponin elevations have been reported in a substantial proportion of stroke patients, with prevalence rates ranging from 27% to 34% (9). These elevations have been consistently linked to heightened mortality in various stroke subtypes, encompassing IS, Intracerebral hemorrhage (ICH), and subarachnoid hemorrhage (SAH). Nevertheless, previous meta-analyses (6, 10–17) have failed to definitively establish troponin elevation as an independent and unequivocal prognostic factor in the context of acute stroke patients. As a result, the debate concerning the predictive utility of elevated cardiac troponin levels in the stroke population continues to be a subject of scientific scrutiny. Therefore, we aimed to conduct a systematic review and meta-analysis, aptly named “Elevated Troponin Levels as a Mortality Predictor in Acute Stroke,” in order to explore the utility of cTn in predicting mortality following acute stroke.

## Methods

### Search strategy

The Cochrane Handbook (version 5.1.0) was used to conduct a systematic review and meta-analysis (18). The preferred reporting items for systematic reviews and meta-analyses (PRISMA) were followed throughout the systematic review’s creation (19). Electronic searches were conducted in databases including PubMed, EMbase, and Trip Databases up to 30th September 2023. Additionally, the reference list of retrieved studies and previous meta-analyses, was manually search for collecting more relevant studies often missed while performing the electronic search. The search aimed to identify studies the prediction of cTn level with the mortality rate in patient with acute stroke. The search utilized a set of specific keywords, including (“Troponin” OR “Cardiac Troponin” OR “cTnT” OR “cTnI” OR “Troponin I” OR “Troponin T” OR “High Sensitive Troponin” OR “High Sensitive Troponin I” OR “hTnI” OR “High Sensitive Troponin T” OR “hTnT”) AND (“Stroke” OR “Brain Stroke” OR “Acute Stroke” OR “Ischemic Stroke” OR “Cerebral Infarction” OR “Intracerebral Hemorrhage” OR “Hemorrhagic Stroke” OR “Subarachnoid Hemorrhage” OR “Aneurysmal Subarachnoid Hemorrhage”) AND (“Mortality” OR “Mortality Rate” OR “Death” OR “Death Rate” OR “Prognosis” OR “Outcome”).

### Selection criteria

*Inclusion Criteria:*

1. Published observational studies, including prospective cohort and retrospective cohort studies that investigate the predictive utility of Cardiac Troponin (cTn) as a biomarker for mortality in patients with acute stroke.
2. Studies that report data related to either in-hospital mortality and/or last follow-up mortality as endpoints.
3. Studies that provide data on cTn assay results at least for a single time point during the course of the study.
4. Studies involving human participants.

*Exclusion Criteria:*

1. Case reports, case series, review articles, gray literature, and editorials.
2. Studies not reporting relevant outcomes.
3. Unavailability of full-texts.

### Data extraction

Two independent authors (“A” and “MS”) conducted a meticulous evaluation of the identified articles to assess their eligibility for inclusion in our study. Upon the initial screening, the authors proceeded to a comprehensive examination of the full-text articles to validate their eligibility and to systematically extract relevant data. To facilitate this process, a standardized data extraction form was utilized, ensuring uniformity and thoroughness in data collection process. The following data was extracted from studies: First Author’s Name, Published year, Country, Study design, Study period, Sample size of elevated cTn and normal cTn groups, source, cTn cut-off value, cTn assessment time point, cTn type, cTn estimation method, In-hospital mortality, Last follow-up mortality outcome measured. The values of cTn levels were reported with different units in the included studies and were converted to similar units for analysis purpose using online unit conversion tools.^1^,^2^ Any disagreement were resolved through discussion with the corresponding author.

### Quality assessment

The quality of the included studies was assessed using the Newcastle-Ottawa Scale (NOS) for observational cohort studies (20). The NOS evaluates study quality across three domains: selection of participants, comparability of groups, and ascertainment of outcomes. Studies were assigned scores ranging from 0 to 9, with higher scores indicating higher quality. Any discrepancies in quality scores were resolved through consensus with the corresponding author.

### Publication bias

To assess publication bias, we employed a funnel plot analysis (21). Egger’s regression test was used to ascertain the asymmetry of funnel plots (22).

### Statistical analysis

A fixed/random-effects model was used to calculate the pooled risk ratio (RR) with 95% confidence interval (CI). Heterogeneity was calculated with the I^2^ statistic. The heterogeneity was considered as significant in case of I^2^ more than 50% for which random effects model was applied, on the other hand, if I^2^ was less than 50%, then fixed-effect model was applied. Sensitivity analysis was performed by sequentially omitting a single study in each turn, to validate the pooled observed effect. In order to ascertain the sufficiency of our sample size and the statistical power of our meta-analysis, we conducted Trial Sequential Analysis (TSA). Tests were considered statistically significant at a *p*-value less than 0.05. All statistical analyses were carried out using STATA, version 12.0 (Stata Statistical Software, Release 12; StataCorp LP, College Station, TX).

## Results

### Search results and study characteristics

**Figure 1:**
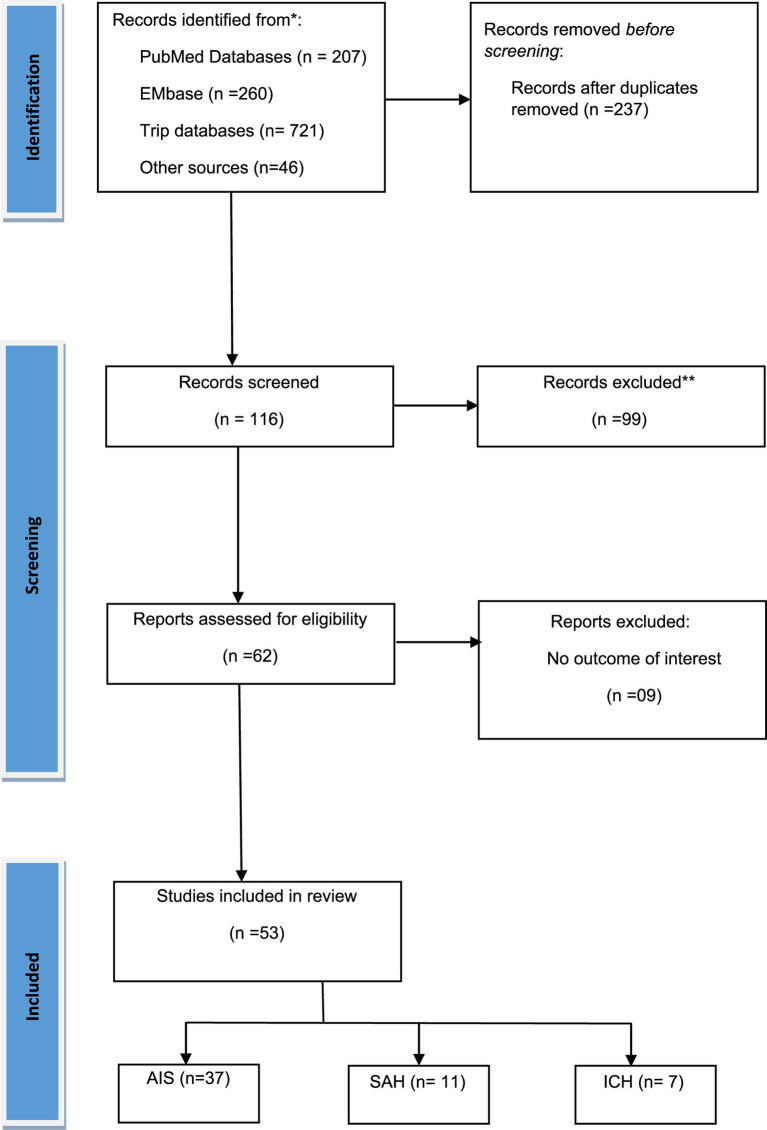
PRISMA flow diagram for the selection of studies and specific reasons for exclusion from the present meta-analysis.

Figure 1 illustrates the PRISMA flow diagram detailing the inclusion and exclusion criteria for studies in our systematic review and meta-analysis. Initially, 1,234 studies were identified across three databases and from other sources. After removing duplicates, 237 articles remained, and following further exclusions, 62 full-text articles were assessed for eligibility. Finally, 53 studies (23–73) were included that examined the role of elevated troponin for predicting mortality in acute stroke. Among these studies, 37 studies focused on AIS patients (23–59), 11 on SAH patients (28, 53, 60–68), and 7 on ICH patients (28, 53, 69–73). The AIS studies involved 3,606 patients with elevated troponin and 14,099 patients with normal troponin, the SAH studies involved 450 patients with elevated troponin and 980 patients with normal troponin, and the ICH studies involved 1,041 patients with elevated troponin and 1,259 patients with normal troponin. Geographically, 24 AIS studies were conducted in Caucasian regions (23–38, 41, 42, 44, 49, 50, 53, 55, 56), and 13 in Asian regions (39, 40, 43, 45–48, 51, 52, 54, 57–59). For SAH, 10 studies were in Caucasian regions (28, 53, 60–66, 68), with only 1 in an Asian region (67). In ICH, 4 studies were in Caucasian regions (28, 53, 69, 72), and 3 in Asian regions (70, 71, 73). Two studies (28, 53) were common for AIS, SAH, and ICH subjects. The publication years ranged from 2000 to 2023, and the sample sizes varied from 43 to 1,718, with detailed baseline characteristics provided in Table 1.

**Table 1:** Baseline characteristics for the included studies investigating for elevated Troponin level in predicting mortality for acute stroke patients.

S. no.	References	Country	Study design	Study period	ETP(*n*)	NTP (*n*)	Cutoff value	cTn assessment timepoint	Type of cTn	Estimation method	Source	Last follow-up duration	Outcome measured
**Acute ischemic stroke**													
1.	James et al. (23)	New Zealand	PCS	July 1997–March 1998	30	151	>0.1 ug/l	Within 72 h of symptom	cTnT	ELISA	Serum	NR	Troponin, mortality
2.	Trooyen et al. (24)	Norway	PCS	January 1999–June 1999	40	109	>0.4 μg/L	Within 48 h of symptom	cTnI	MEIA	Serum	NR	Neurological outcomes, mortality
3.	Angelontanio et al. (25)	Italy	PCS	February 2001–January 2002	18	277	>0.01 ng/mL	Within 24 h of symptom	cTnI	RIA	NR	NR	Mortality, non-fatal MI event, non-fatal cardiopulmonary event
4.	Barber et al. (26)	UK	PCS	NR	45	177	>0.2 μg/L	Within 24 h of symptom	cTnI	CIA	Serum	30 days	Death or dependency (Rankin > 2)
5.	Jensen et al. (27)	Denmark	PCS	August 2003–October 2004	25	219	>0.03 μg/L	Within 7 days after symptom	hs-cTnT	ELISA	NR	19 months	All-cause in-hospital mortality
6.	Sandhu et al. (28)	USA	PCS	NR	23	138	>0.4 ng/mL	Within 24 h of symptom	cTnI	NR	Serum	NR	In-hospital mortality
7.	Hajdinjak et al. (29)	Slovenia	PCS	June 2006–May 2010	16	90	>0.04 μg/L	Within 24 h of symptom	cTnT	ELISA	NR	NR	In-hospital mortality
8.	Scheitz et al. (30)	Germany	RCS	October 2009–October 2010	103	612	>0.03 μg/L	Within 7 days after symptom	cTnI	ELISA	NR	NR	Unfavorable outcome, Major neurologic improvement, In-hospital mortality
9.	Jensen et al. (31)	USA	PCS	August 2003–October 2004	65	128	>14 ng/L	Within 24 h of symptom	hs-cTnT	CIA	NR	4.4 years	All-cause mortality
10.	Boire et al. (32)	Canada	PCS	October 2006–November 2010	46	362	>0.03 ug/L	Within 72 h of symptom	cTnI	NR	NR	90 days	Death, myocardial Infarction, mRS score, recurrent stroke
11.	Stahrenberg et al. (33)	Germany	PCS	March 2009–February 2010	98	99	≥6.15 pg./mL	Within 24 h of symptom	hs-cTnT	ELISA	Plasma	12 months	Vascular events, all-cause mortality
12.	Scheitz et al. (34)	Germany	PCS	February 2011–Feb ruary2013	308	409	>14 ng/L	Within 48 h of symptom	cTnT	ELISA	NR	NR	Unfavorable neurological outcome and in-hospital mortality
13.	Lasek-Bal et al. (35)	Poland	PCS	2013–2014	104	964	> 0.014 ng/mL	Within 72 h of symptom	cTnI	ELISA	Serum	30 days	Mortality, mRS 4–6
14.	Faiz et al. (36)	Norway	RCS	June 2009–May 2010	156	131	>14 ng/L	within 24 h of symptom	hs-cTnT	ELISA	Serum	NR	In-hospital mortality
15.	Raza et al. (37)	USA	RCS	2008–2010	17	183	>0.40 ng/mL	Within 24 h of symptom	cTnI	CIA	Serum	2 years	Long term cardiac adverse event, all cause death
16.	Batal et al. (38)	USA	PCS	December 2008–November 2010	309	1,409	>0.1 μg/L	Within 24 h of symptom	cTnI	CIA	Serum	3 years	Mortality
17.	Maoz et al. (39)	Israel	RCS	1 year	35	177	>0.03 μg/L	NR	hs-cTnT	NR	Serum	NR	NIHSS and mortality
18.	Su et al. (40)	Taiwan	RCS	August 2010–March 2015	146	725	>0.01 μg/L	Within 48 h of symptom	cTnI	ELISA	Serum	NR	poor outcome and in-hospital mortality
19.	Budincevic et al. (41)	Croatia	RCS	2007–2010	10	188	>0.5 ug/L	Within 24 h of symptom	cTnI	NR	NR	NR	Unfavorable outcome, death
20.	Peddada et al. (42)	USA	RCS	May 2008–December 2012	199	946	>0.120 ng/mL	Within 7 days after symptom	cTnI	CIA	Serum	NR	NIHSS and mortality
21.	Akpinar et al. (43)	Turkey	RCS	NR	11	53	>0.014 ng/mL	Within 48 h of symptom	cTnT	CIA	Serum	Mean: 9.6 days	In-hospital mortality
22.	Wrigley et al. (44)	USA	RCS	2010	295	1,082	between 40 and 50 ng/L	Within 24 h of symptom	cTnT and cTnI	NR	Serum	3 Year	Long-term mortality, Structural cardiac disease
23.	Ahn et al. (45)	Korea	RCS	May 2007–December 2014	166	1,526	>0.04 ng/mL	Within 48 h of symptom	cTnI	CMIA	Serum	33 months	Long-term mortality
24.	Fathy et al. (46)	Egypt	PCS	March 2016–December 2016	14	60	>0.01 μg/L.	Within 48 h	cTnI	ELISA	Serum	NR	Poor short-term outcome and in-hospital mortality
25.	He L. et al. (47)	China	RCS	May 2012–December 2017	118	398	≥14 ng/L	Within 72 h	hs-cTnT	CIA	Serum	3 months	Death, Major disability
26.	Ahn et al. (48)	Korea	PCS	2013–2015	145	947	≥0.040 ng/mL	Within 48 h	cTnI	CIA	Serum	18 months	Adverse long-term outcomes, mortality
27.	He M. et al. (49)	USA	RCS	January 2013–December 2016	25	58	0.6 ng/mL	Within 48 h	cTn	NR	NR	1 year	All-cause mortality, AMI, AIS, and all-cause re-hospitalization
28.	Terceno et al. (50)	Spain	PCS	NR	42	68	≥14 ng/L	Within 24 h of symptom	hs-cTnT	NR	Plasma	3 months	Mortality, mRS, NIHSS
29.	Sui et al. (51)	China	PCS	January 2017–February 2018	65	176	>14 ng/L	Within 24 h of symptom	hs-cTnT	NR	Serum	90 days	Mortality, disability
30.	Cao et al. (52)	China	RCS	January 2015–November 2017	85	58	>14 ng/L	Within 48 h of symptom	hs-cTnT	CIA	Serum	3 months	Poor outcome and mortality
31.	Alkhachroum et al. (53)	USA	RCS	January 2013–April 2015	197	621	>0.04 ng/mL	Within 48 h of symptom	cTnI	NR	Serum	NR	Disease severity, mortality, functional outcome and discharge disposition
32.	Thapa et al. (54)	Nepal	PCS	NR	8	93	> 0.034 ng/mL	Within 48 h of symptom	hs-cTnI	CIA	Serum	NR	Poor outcomes and in-hospital mortality
33.	Scheitz et al. (55)	Germany	PCS	January 2010–June 2013	220	342	>14 ng/L	Within 7 days of symptom	hs-cTnT	CIA	Serum	3 year	Recurrent stroke, myocardial infarction, and all-cause death
34.	Nageeb et al. (56)	Egypt	PCS	January 2017–January 2019	13	59	≥0.01 μg/L	Within 24 h after onset	cTnI	ELISA	Serum	3 months	Death, disability, neurological improvement
35.	Miraj et al. (57)	Bangladesh	PCS	NR	8	92	>0.04 ng/mL	Within 7 days after symptom	cTnI	NR	Serum	NR	Poor outcome and in-hospital mortality
36.	Chen et al. (58)	China	PCS	February 2016–November 2020	90	264	>0.03ug/L	within 24 h after onset	hs-cTnI	CIA	NR	90 days	hscTnI, mortality
37.	Kim et al. (59)	Korea	RCS	August 2014–July 2017	311	708	20.7 ng/L for men and 16.1 ng/L for women	within 24 h of symptom	hs-cTnI	CIA	NR	22.5 months	Mortality and cardiac and cerebrovascular events
**Subarachnoid hemorrhage**													
38.	Deibert et al. (60)	USA	PCS	January 1998–August 2000	12	31	≥1.4 ug/L	On admission	cTnI	NR	Serum	NR	Home, acute rehabilitation, nursing home and death
39.	Naidech et al. (61)	USA	PCS	November 1998–October 2002	46	81	>0 to 0.5 ug/L	On admission	cTnI	NR	Serum	3 months	Functional disability or death
40.	Sandhu et al. (28)	USA	PCS	NR	20	76	>0.4 ng/mL	On admission	cTnI	NR	Serum	NR	In-hospital mortality
41.	Ramappa et al. (62)	USA	PCS	January 1999–December 2003	31	52	≥2.0 ng/mL	On admission	cTnI	NR	Serum	NR	Worse neurological outcome and in-hospital mortality
42.	Gupte et al. (63)	USA	RCS	August 2006–June 2009	47	157	>0.5 ng/mL	On admission	cTnI	NR	NR	NR	In-hospital mortality
43.	Duello et al. (64)	USA	RCS	March 2011–July 2013	36	139	>0.10 ng/mL	On admission	cTnT	NR	NR	1 month	Mortality
44.	Guette et al. (65)	France	PCS	October 2012–August 2015	76	61	>22 ng/L	within 72 h of symptom onset	hs-cTnT	ELISA	Plasma	3 months	Sensitivity and specificity with corresponding 95% CIs.
45.	Alkhachroum et al. (53)	USA	RCS	January 2013–April 2015	48	75	>0.04 ng/mL	On admission	cTnI	NR	Serum	NR	Disease severity, Mortality, functional outcome and discharge disposition
46.	Akkermans et al. (66)	Netherland	PCS	November 2005–February 2008	43	116	>12 ng/L	NR	cTnI	NR	Serum	1 year	Myocardial infarction, cardiac death, all-cause mortality
47.	Lin et al. (67)	China	PCS	January 2016–December 2017	55	158	>0.016 ng/mL	On admission	cTnI	NR	NR	34.3 months	Major adverse cardiac events, long-term neurological outcomes, and mortality
48.	Anetsberger et al. (68)	Germany	PCS	March 2013–December 2015	36	34	>0.007 μg/L	On admission	cTnT	ELISA	NR	3 months	Functional outcome and mortality
**Intracerebral hemorrhage**													
49.	Hays et al. (69)	USA	RCS	January 2001–January 2005	38	84	>0.10 ng/mL	within 24 h	cTnI	RIA	Serum	NR	In-hospital mortality
50.	Sandhu et al. (28)	USA	PCS	NR	14	80	>0.4 ng/mL	within 24 h	cTnI	NR	Serum	NR	In-hospital mortality
51.	Chung et al. (70)	South Korea	RCS	January 2003–December 2007	28	225	>0.01 ng/mL	Within 24 admission hours	cTnT	ELISA	Serum	NR	In-hospital mortality
52.	Xu et al. (71)	China	RCS	NR	66	122	NR	NR	cTnI	NR	NR	NR	In-hospital mortality, GCS < 8, NIHSS >10
53.	Gerner et al. (72)	Germany	PCS	2006–2014	83	437	>0.040 ng/mL	Within 72 h after admission	cTnI	CIA	Plasma	NR	Functional outcome
54.	He et al. (73)	China	RCS	June 2012–December 2015	729	88	≥0.028 ng/mL	NR	cTnI	CIA	Serum	NR	In-hospital mortality and mRS
55.	Alkhachroum et al. (53)	USA	RCS	January 2013–April 2015	83	223	>0.04 ng/mL	On admission	cTnI	NR	Serum	NR	Disease severity, mortality, functional outcome and discharge disposition

RCS, Retrospective Cohort Study; PCS, Prospective Cohort Study; cTnI, Cardiac troponin I; cTnT, Cardiac troponin T; hs-cTnT, High-sensitive cardiac Troponin T; hs-cTnI, High-sensitive cardiac Troponin I; NIHSS, National Institute of Health Stroke Scale; GCS, Glasgow Coma Scale; mRS, Modified Rankin Scale; NR, not reported; ELISA, enzyme-linked immunosorbent assay; CIA, chemiluminescence immunoassay; RIA, radioimmunoassay; MEIA, Microparticle Enzyme Immunoassay; CMIA, chemiluminescent microparticle immunoassay.

Regarding predictive mortality outcomes, 15 studies with AIS patients (23–25, 28–30, 34, 36, 39–43, 54, 57), 6 with SAH (28, 53, 60, 62, 63, 67), and 7 with ICH (28, 53, 69–73) reported data for In-hospital mortality. For last follow-up mortality, 22 AIS studies (26, 27, 31–35, 37, 38, 44–53, 56, 58, 59) and 6 SAH studies (61, 64–68) provided relevant data. Only a single study (67) reported data both In-hospital mortality and last follow-up mortality for SAH subjects. The majority of studies included in our review received high ratings on the NOS Scale, indicating strong methodological quality (Table 2). Each study’s total score, ranging from 6 to 9, reflects its overall quality, supporting the reliability and validity of the evidence presented in this review.

**Table 2:** Quality assessment of included studies based on Newcastle-Ottawa Scale (NOS).

	Selection					Comparability	Outcome			Result
S. no.	References	Representative of the exposed cohort	Selection of the non-exposed cohort	Ascertainment of exposure	Demonstration that outcome was not present at study start	Comparability of cohorts based on design or analysis	Assessment of outcome	Enough follow up period	Adequacy of follow up	Total score
**Acute ischemic stroke**										
1.	James et al. (23)	1	1	1	1	1	1		1	7
2.	Trooyen et al. (24)	1	1	1	1	1	1			6
3.	Angelontanio et al. (25)	1	1	1	1	2	1	1	1	9
4.	Barber et al. (26)	1	1	1	1	2	1	1	1	9
5.	Jensen et al. (27)	1	1	1	1	2	1	1	1	9
6.	Sandhu et al. (28)	1	1	1	1	1	1			6
7.	Hajdinjak et al. (29)	1	1	1	1	2	1			7
8.	Scheitz et al. (30)	1	1	1	1	2	1			7
9.	Jensen et al. (31)	1	1	1	1	2	1	1		8
10.	Boire et al. (32)	1	1	1	1	2	1	1	1	9
11.	Stahrenberg et al. (33)	1	1	1	1	2	1	1	1	9
12.	Scheitz et al. (34)	1	1	1	1	2	1			7
13.	Lasek-Bal et al. (35)	1	1	1	1	1	1			6
14.	Faiz et al. (36)	1	1	1	1	2	1			7
15.	Raza et al. (37)	1	1	1	1	2	1	1	1	9
16.	Batal et al. (38)	1	1	1	1	2	1	1		8
17.	Maoz et al. (39)	1	1	1	1	2	1			7
18.	Su et al. (40)	1	1	1	1	2	1			7
19.	Budincevic et al. (41)	1	1	1	1	1	1			6
20.	Peddada et al. (42)	1	1	1	1	2	1			7
21.	Akpinar et al. (43)	1	1	1	1	1	1	1		7
22.	Wrigley et al. (44)	1	1	1	1	2	1			7
23.	Ahn et al. (45)	1	1	1	1	2	1	1	1	9
24.	Fathy et al. (46)	1	1	1	1	2	1			7
25.	He L. et al. (47)	1	1	1	1	2	1	1		8
26.	Ahn et al. (48)	1	1		1	2	1	1		7
27.	He M. et al. (49)	1	1	1	1	2	1	1	1	9
28.	Terceno et al. (50)	1	1	1	1	2	1	1	1	9
29.	Sui et al. (51)	1	1	1	1	2	1	1	1	9
30.	Cao et al. (52)	1	1	1	1	2	1	1	1	9
31.	Alkhachroum et al. (53)	1	1	1	1	2	1			7
32.	Thapa et al. (54)	1	1	1	1	2	1			7
33.	Scheitz et al. (55)	1	1	1	1	2	1	1	1	9
34.	Nageeb et al. (56)	1	1	1	1	2	1	1	1	9
35.	Miraj et al. (57)	1	1	1	1	1	1			6
36.	Chen et al. (58)	1	1	1	1	2	1	1	1	9
37.	Kim et al. (59)	1	1	1	1	2	1	1	1	9
**Subarachnoid hemorrhage**										
38.	Deibert et al. (60)	1	1	1	1	1	1	1	1	8
39.	Naidech et al. (61)	1	1	1	1	1	1	1		7
40.	Sandhu et al. (28)	1	1	1	1	1	1			6
41.	Ramappa et al. (62)	1	1	1	1	2	1	1	1	8
42.	Gupte et al. (63)	1	1	1	1	2	1			7
43.	Duello et al. (64)	1	1	1	1	2	1	1	1	9
44.	Guette et al. (65)	1	1	1	1	2	1	1	1	9
45.	Alkhachroum et al. (53)	1	1	1	1	2	1			7
46.	Akkermans et al. (66)	1	1	1	1	1	1	1	1	8
47.	Lin et al. (67)	1	1	1	1	2	1	1	1	9
48.	Anetsberger et al. (68)	1	1	1	1	2	1	1	1	9
**Intracerebral hemorrhage**										
49.	Hays et al. (69)	1	1	1	1	2	1			7
50.	Sandhu et al. (28)	1	1	1	1	1	1			6
51.	Chung et al. (70)	1	1	1	1	2	1			7
52.	Xu et al. (71)	1	1	1	1	2	1	1	1	9
53.	Gerner et al. (72)	1	1	1	1	2	1	1	1	9
54.	He et al. (73)	1	1	1	1	2	1			7
55.	Alkhachroum et al. (53)	1	1	1	1	2	1			7

### In-hospital mortality

**Figure 2:**
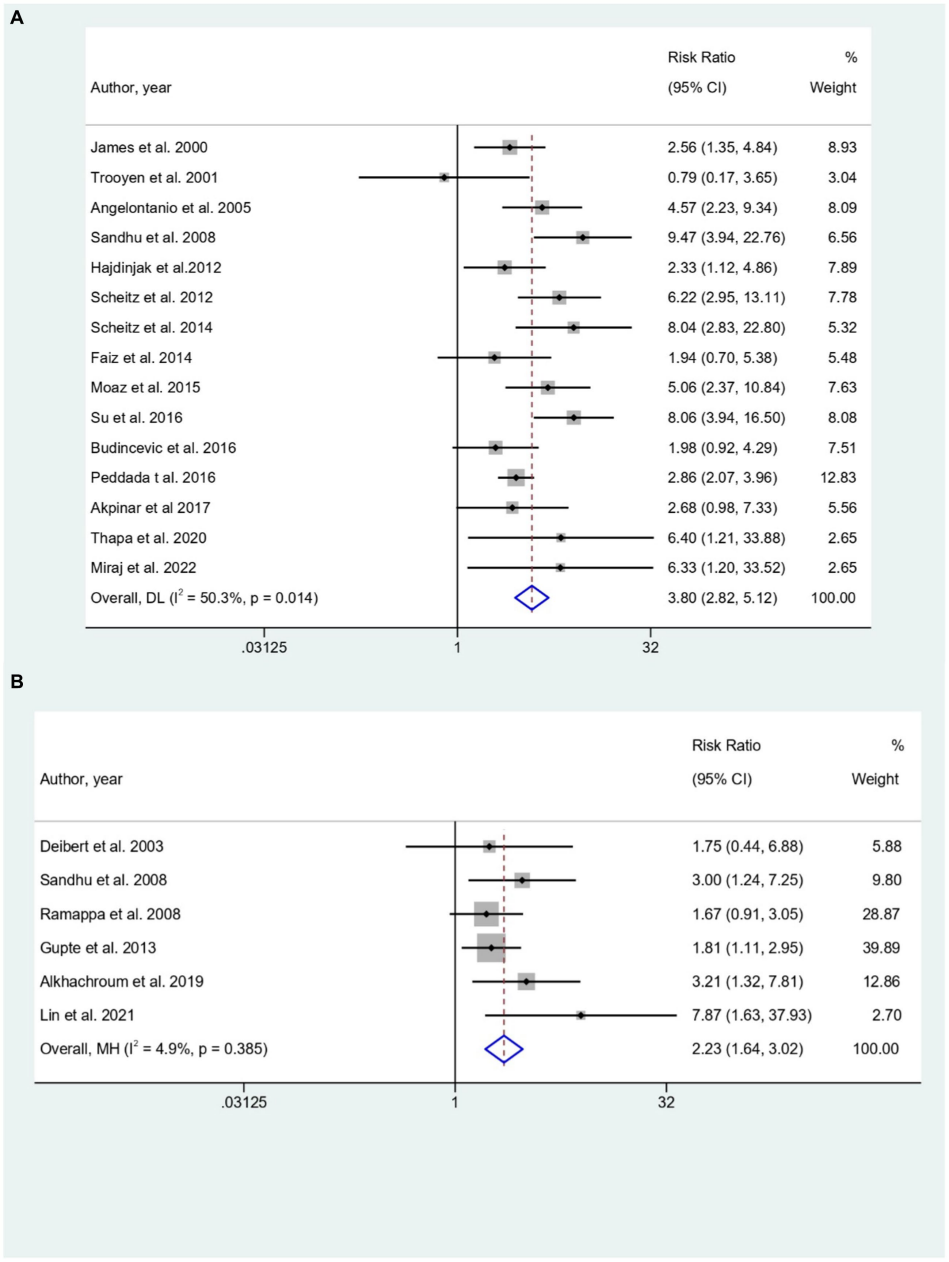

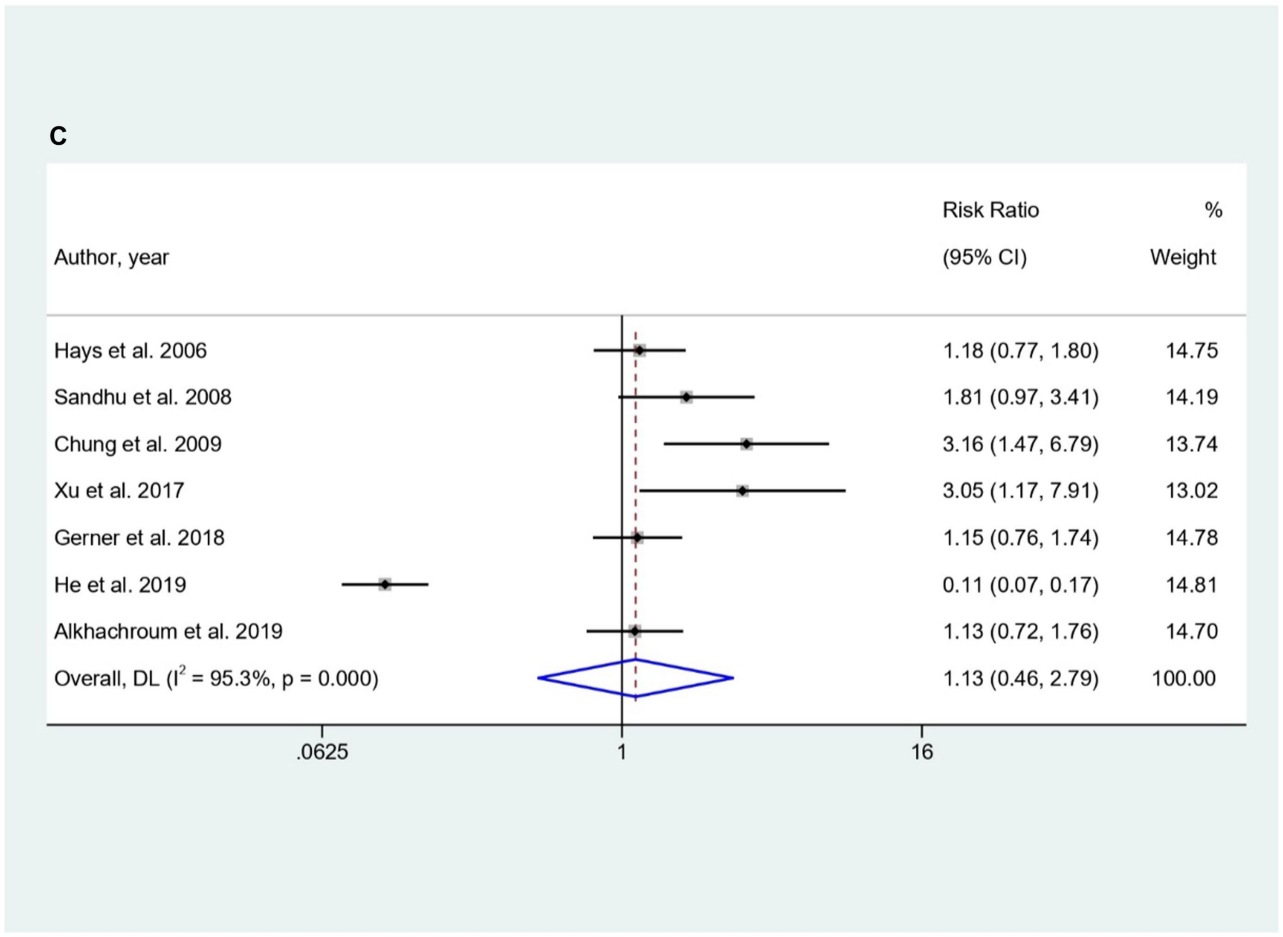
**(A–C)** Forest plot for the prediction of In-hospital mortality in **(A)** AIS, **(B)** SAH, and **(C)** ICH with respect to elevated cTn Levels.

Elevated cTn levels were significantly showed a higher predictive risk for In-hospital mortality in both AIS (RR = 3.80, 95% CI; 2.82 to 5.12; Figure 2A) as well as SAH (RR = 2.23, 95% CI; 1.64 to 3.02; Figure 2B). However, no significant predictive risk between elevated cTn levels and in-hospital mortality for ICH patients (RR = 1.13, 95% CI: 0.46 to 2.79; Figure 2C).

Further subgroup analysis based on type of cTn, study design, Troponin cut-off value, and estimation method of troponin showed diverse patterns. Specifically for AIS subgroup analysis based on type of troponin, cTnI had a higher prediction risk (RR: 3.93; 95% CI: 2.34 to 6.60) than cTnT (RR: 3.63; 95% CI: 2.24 to 5.88) and hs-cTnT (RR: 3.34; 95% CI: 1.32 to 8.47) while hscTnI had (RR: 6.40; 95% CI: 1.21 to 33.88; Supplementary Table S1A). Based on study design, prospective cohort studies (PCS) had a higher risk (RR: 4.00; 95% CI: 2.43 to 6.57) compared to retrospective cohort studies (RCS) [RR: 3.66; 95% CI: 2.45 to 5.47]. Troponin cut-off values also showed variation, with 0.01 to 0.05 μg/L at RR: 4.55 (95% CI: 3.29 to 6.29) and 0.1 to 0.5 μg/L at RR: 2.86 (95% CI: 1.70 to 4.83). Assessment time-points within 24, 48, and 72 h had different risks.

The choice of troponin estimation methods significantly influenced risk, with Enzyme-Linked Immunosorbent Assay (ELISA) resulting in RR: 3.56 (95% CI: 2.07 to 6.12), Radioimmunoassay (RIA) at RR: 4.57 (95% CI: 2.23 to 9.34), and chemiluminescence immunoassay (CIA) at RR: 2.92 (95% CI: 2.16 to 3.96). Prediction risks varied at assessment time-points within 24, 48, and 72 h, but stability was observed for studies examining troponin levels within 24 h after Acute Ischemic Stroke (AIS). For SAH patients, subgroup analysis for showed that cTnI had a higher predictive risk for In-hospital mortality (RR = 2.23; 95% CI: 1.64 to 3.02). Both PCS (RR = 2.31; 95% CI: 1.49 to 3.58) and RCS (RR = 2.15; 95% CI: 1.40 to 3.31) also showed higher predictive role of elevated troponin for the In-hospital mortality in SAH patients (Supplementary Table S1B). For ICH, subgroup analysis revealed non-significant predictive risk between elevated cTn levels and in-hospital mortality for ICH patients (Supplementary Table S1C). Only significant association for the assessment time point of troponin levels within 24 h and within 48 h after ICH (RR = 1.76; 95% CI: 1.01 to 3.06) and (RR = 3.05; 95% CI: 1.17 to 7.91) respectively were observed (Supplementary Table S1C).

### Last follow-up mortality

**Figure 3:**
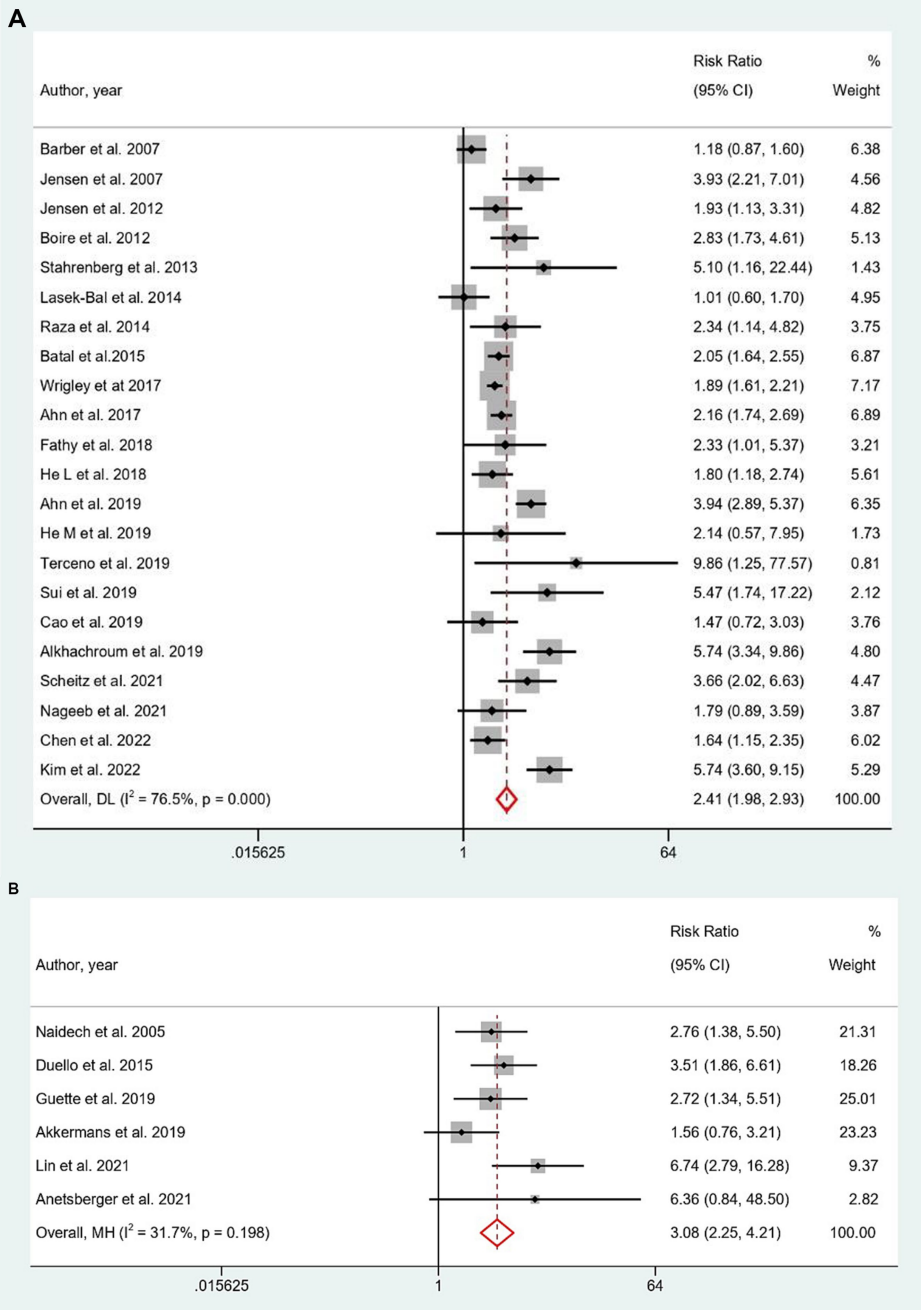
**(A,B)** Forest plot for the prediction of last follow-up mortality in **(A)** AIS and **(B)** SAH with respect to elevated cTn Levels.

Elevated cTn levels were also significantly showed a higher predictive risk for last follow-up mortality in both AIS (RR = 2.41, 95% CI: 1.98 to 2.93; Figure 3A) and SAH (RR = 3.08, 95% CI: 2.25 to 4.21; Figure 3B). Subgroup analysis showed hs-cTnT stood out with a significantly higher prediction risk (RR: 6.02; 95% CI: 2.60 to 13.93) in AIS, compared to cTnT and cTnI (Supplementary Table S2A). Both prospective and retrospective study designs, indicated an elevated predictive role of elevated troponin for last follow-up mortality in AIS and SAH subgroup analysis (Supplementary Tables S2A,B). Predictive risk remained stable for studies examining troponin levels within 24 h after AIS and SAH. Troponin estimation methods varied for AIS studies, with most utilizing CIA method, while for SAH, the predominant method was ELISA. Analysis on last follow-up mortality in ICH group was not feasible as only a single study provided data for it.

### Publication bias

Funnel plot analysis did not reveal any apparent signs of asymmetry. The distribution of studies around the estimated effect appeared to be even, suggesting the absence of significant publication bias that could impact the results. Funnel plots for the in-hospital mortality in patients with AIS (*p* = 0.45), SAH (*p* = 0.10), and ICH (*p* = 0.19), and last follow-up mortality for AIS (*p* = 0.13), and SAH (*p* = 0.42), are represented in Supplementary Figures S1A–C, S2A,B.

### Sensitivity analysis

Sensitivity analysis indicated that the pooled risk estimates for in-hospital mortality in patients with AIS, SAH, and ICH was not significantly affected by the removal of any individual study (Supplementary Figures S3A–C). Similarly, the pooled risk estimates for last follow-up mortality in patients with AIS and SAH was not significantly affected by the removal of any individual study (Supplementary Figures S4A,B). Our findings suggest that the results of the meta-analysis are robust and are not driven by any single study which provides additional confidence in the validity of the findings.

### Trial sequential analysis

**Figure 4:**
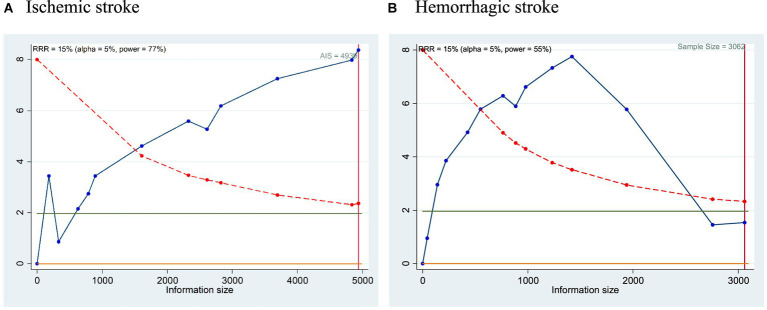
**(A,B)** TSA plot for the prediction of mortality in **(A)** Ischemic stroke and **(B)** Hemorrhagic stroke with respect to elevated cTn Levels.

Our Trial sequential analysis (TSA) showed a very promising strength with 77% power with total sample size of 4,939 AIS patients and 55% power for the studies with 3,062 HS subjects. TSA plot are represented in Figures 4A,B. The high power of the TSA suggests that the meta-analysis is unlikely to be influenced by chance or random fluctuations in the data. Overall, the TSA results provide strong evidence that the meta-analysis is well-powered and that the findings are reliable and generalizable. This reinforces the importance of considering troponin elevation as a crucial factor in risk stratification and treatment decisions for patients with acute stroke.

## Discussion

Our findings highlights the significance of routinely assessing admission cTn in stroke patients, emphasizing its association with higher mortality rates. Existing literature has consistently reported the association between elevated troponin levels and all-cause mortality across various stroke types (10, 12). Studies have shown that baseline troponin levels predict poor outcomes in both in-hospital and last follow-up scenarios, irrespective of acute coronary syndrome (69, 71, 72). Furthermore, higher blood troponin levels during the acute phase of acute spontaneous ICH are linked with unfavorable outcomes (69, 71, 72). Similar findings are observed in patients with spontaneous subarachnoid hemorrhage, where an increased risk of cerebral ischemia and death is demonstrated (13, 14, 60).

The current meta-analysis supports and reinforces these findings, indicating that elevated troponin levels serve as predictors for both in-hospital and follow-up mortalities for AIS with additional finding of other subtypes of stroke including SAH and ICH. Elevated cTn levels were significantly showed a higher predictive risk for In-hospital mortality and last follow-up mortality in both AIS as well as SAH. However, no significant predictive risk between elevated cTn levels and in-hospital mortality for ICH patients. However, it’s crucial to note that this observation may be influenced by the limited inclusion of only seven studies in the overall analysis.

Previous findings have reported that elevation of cardiac troponin has been reported to occur in ICH patients along with only 1.2% of them died of cardiac causes (69). Although previous studies have been tried to address hypothesis that elevated cardiac troponin might serve as prognostic markers for prediction of adverse clinical events, the results were largely inconsistent and inconclusive with regard to ICH. The previous study demonstrated that elevated troponin levels were associated with higher mortality following ICH (69). Subsequently, the utility of elevated troponin levels for prediction of mortality was confirmed in surgical ICH patients (74), but was not found to be consistently associated with in-hospital mortality in Chinese ICH patients (71). Small sample sizes (less than 240 stroke patients) and ethnic variability probably contribute to the negative results and discrepancies. Similarly in our meta-analysis, only seven ICH studies were included and we observed non-significant association with the elevated level of Troponin.

Approximately 18%–20% (75–77) of ischemic stroke patients present with elevated high-sensitive Troponin T (hsTnT) levels on admission, which can be attributed to various factors including renal failure, chronic heart failure, myocardial infarction (MI), and stress-related cardiomyopathies such as neurogenic stunned myocardium (NSM). NSM, characterized by contraction band necrosis at the cellular level, manifests with elevated hsTnT levels and electrocardiographic abnormalities (78–81). While some patients can be differentiated using ECG and echocardiography, overlap in diagnostic criteria complicates diagnosis for others. Despite common assumptions in stroke centers, attributing elevated hsTnT levels solely to brain or heart origin may not always hold true, as MI patients have an increased risk of stroke (82), even with marginally elevated hsTnT levels, raising questions about predictive value in differentiating NSM from MI (83).

As per the 2023 Guideline for the Management of Patients with SAH by AHA/ASA (84), certain medical parameters, such as BMI, hypertension, hyperglycemia, troponin levels, hyperthermia, peak white blood cell count, C-reactive protein, and high neutrophil counts, have been linked to clinical outcomes in SAH. However, it is emphasized that additional investigation is necessary to determine their prognostic value and influence on treatment outcomes. Furthermore, the guideline underscores the active exploration of novel biomarkers, encompassing imaging, serum, and cerebrospinal fluid (CSF), in the field of SAH. Ongoing research incorporating advanced proteomic, genomic, and other biological marker methods, in conjunction with existing clinical, radiographic, and physiological monitoring data, is deemed essential. These efforts aim to shed light on the potential use of biomarkers for prognosis and interventions, ultimately contributing to improved outcomes in patients with SAH.

The COVID-19 pandemic has placed unprecedented strain on stroke services globally, disrupting healthcare delivery and raising concerns about its impact on stroke outcomes (85). Despite these challenges, our meta-analysis did not identify any explicit instances linking the pandemic with influential impacts on stroke management or patient outcomes across included studies. This highlights the need for further research to understand the nuanced interplay between COVID-19 and stroke care delivery, while also emphasizing the importance of developing adaptive strategies and fostering interdisciplinary collaborations to mitigate the pandemic’s adverse effects on stroke services and ensure optimal patient care.

Clinical decision-making regarding troponin elevations in stroke patients is a multifaceted challenge that requires careful consideration of various factors. While elevated troponin levels in stroke patients can indicate myocardial injury, termed “troponitis,” it’s essential to recognize that other contributing factors such as stress, sepsis, or renal dysfunction may also lead to troponin elevation, particularly in the acute phase of a stroke (86). Therefore, clinicians must conduct a thorough evaluation to differentiate between myocardial infarction and other causes of troponin elevation (87). This evaluation typically involves a comprehensive clinical assessment, including medical history, physical examination, electrocardiography, and imaging studies such as echocardiography or cardiac MRI (88).

Additionally, clinicians may utilize risk stratification tools or algorithms specific to stroke patients with troponin elevations to guide further diagnostic and therapeutic interventions. Collaboration between neurologists, cardiologists, and other specialists is crucial in developing a cohesive management plan tailored to the individual patient’s needs, balancing the risks and benefits of interventions such as antiplatelet therapy, anticoagulation, or coronary angiography (89). Ultimately, the decision-making process should prioritize patient safety, optimizing outcomes, and improving overall care in stroke units. Ongoing research and consensus guidelines from professional societies play a vital role in informing evidence-based practices and advancing our understanding of troponin elevations in stroke patients.

### Study limitations

While the study has strengths, it acknowledges limitations, including: (1) using a single baseline cTn assessment time point and non-standardized measurement procedures, potentially introducing misclassification; (2) lacking information on important confounders like infarction locations, stroke severity, time from symptom onset, and type of recanalization therapies, which could bias the relationship between elevated cTn and mortality; (3) absence of data on functional outcomes like the modified Rankin Scale; and (4) variations in cut-off values, assay methods, and follow-up periods across individual studies contributed to high heterogeneity from pooled analysis by troponin assays, complicating result interpretation.

## Conclusion

Elevated troponin levels can serve as a promising predictive marker for both in-hospital and last follow-up mortality in AIS and SAH patients but not in ICH patients. Further prospective studies are needed to validate our findings along with exploring the preventive management of mortality in acute stroke settings.

## Data availability statement

The original contributions presented in the study are included in the article/Supplementary material, further inquiries can be directed to the corresponding author.

## Author contributions

AG: Writing – review & editing, Writing – original draft, Methodology, Data curation. MS: Investigation, Writing – review & editing, Methodology, Data curation, Project administration. PK: Writing – review & editing, Writing – original draft, Supervision, Software, Formal analysis, Conceptualization.

## Footnotes

^1^ http://www.endmemo.com

^2^ https://www.unitconverters.net/
