# Supplementary material for: Elevated troponin levels as a predictor of mortality in patients with acute stroke: a systematic review and meta-analysis
Source: Front Neurol. 2024 Mar 25;15:1351925. doi: 10.3389/fneur.2024.1351925 (PMC10999611; doi:10.3389/fneur.2024.1351925)
Supplement: Supplementary file 1 [file Table_1.docx]

**Supplementary Table legends**

**Table-S1(A-C):** Summary of estimates based on subgroup analysis of In-hospital mortality in (A) AIS (B) SAH and (C) ICH.

**Table-S2 (A-B):** Summary of estimates based on subgroup analysis of last follow-up mortality in (A) AIS and (B) SAH.

**Table-S1 (A):** Summary of estimates based on subgroup analysis of In-hospital mortality in AIS.

| **Variables** | **Subgroups items** | **No. of studies** | **Risk Ratio**  **(95% Confidence Interval)** | **Degree of Heterogeneity** | |
| --- | --- | --- | --- | --- | --- |
|  |  |  |  | **I^2^ (%)** | **p* values** |
| Troponin type | cTnT | 5 | 3.63 (2.24 to 5.88) | 43.7 | 0.131 |
|  | cTnI | 7 | 3.93 (2.34 to 6.60) | 67.4 | **0.005** |
|  | hs-cTnT | 2 | 3.34 (1.32 to 8.47) | 54.2 | 0.140 |
|  | hs-cTnI | 1 | 6.40 (1.21 to 33.88) | 0.0 | - |
| Study design | PCS | 8 | 4.00 (2.43 to 6.57) | 51.3 | **0.045** |
|  | RCS | 7 | 3.66 (2.45 to 5.47) | 55.7 | **0.036** |
| Troponin cut-off value | 0.1 to 0.5 µg/l | 5 | 2.86 (1.70 to 4.83) | 63.2 | **0.028** |
|  | 0.01 to 0.05 µg/l | 10 | 4.55 (3.29 to 6.29) | 22.3 | 0.238 |
| Troponin assessment timepoint | Within 24 hrs | 5 | 3.47 (2.82 to 5.12) | 55.2 | 0.063 |
|  | Within 48 hrs | 6 | 4.30 (2.36 to 7.83) | 43.7 | 0.114 |
|  | Within 72 hrs | 4 | 3.77 (2.01 to 7.08) | 61.1 | 0.076 |
| Estimation Method | ELISA | 7 | 3.56 (2.07 to 6.12) | 64.3 | **0.010** |
|  | RIA | 1 | 4.57 (2.23 to 9.34) | 0.0 | - |
|  | CIA | 3 | 2.92 (2.16 to 3.96) | 0.0 | 0.639 |
|  | NR | 4 | 4.68 (2.25 to 9.75) | 59.2 | **0.062** |
| **Overall** | | | 3.80 (2.82 to 5.12) | 50.3 | **0.014** |

***** **Bold values represent statistically significant heterogeneity (p-value < 0.05)**

**Table-S1 (B):** Summary of estimates based on subgroup analysis of In-hospital mortality in SAH.

| **Variables** | **Subgroups items** | **No. of studies** | **Risk Ratio**  **(95% Confidence Interval)** | **Degree of Heterogeneity** | |
| --- | --- | --- | --- | --- | --- |
|  |  |  |  | **I^2^ (%)** | **p* values** |
| Troponin type | cTnI | 6 | 2.23 (1.64 to 3.02) | 4.09 | 0.385 |
| Study design | PCS | 4 | 2.31 (1.49 to 3.58) | 24.0 | 0.267 |
|  | RCS | 2 | 2.15 (1.40 to 3.31) | 21.1 | 0.260 |
| Troponin cutoff value | 1 to 2 µg/l | 2 | 1.68 (1.68 to 2.92) | - | 0.951 |
|  | 0.1 to 0.5 µg/l | 2 | 2.04 (1.34 to 3.13) | 69.5 | 0.020 |
|  | 0.01 to 0.05 µg/l | 2 | 4.02 (1.88 to 8.59) | 0.331 | 0.171 |
| Troponin assessment timepoint | Within 24 hrs | 2 | 2.53 (1.21 to 5.29) | 0.0 | 0.516 |
|  | Within 72 hrs | 1 | 7.87 (1.63 to 37.93) | 0.0 | - |
|  | Within 7 days | 3 | 1.98 (1.40 to 2.81) | 0.0 | 0.453 |
| **Overall** | | | **2.23 (1.64** **to 3.02)** | **4.9** | **0.385** |

***** **Bold values represent statistically significant heterogeneity (p-value < 0.05)**

**Table-S1(C):** Summary of estimates based on subgroup analysis of In-hospital mortality in ICH.

| **Variables** | **Subgroups items** | **No. of studies** | **Risk Ratio**  **(95% Confidence Interval)** | **Degree of Heterogeneity** | |
| --- | --- | --- | --- | --- | --- |
|  |  |  |  | **I^2^ (%)** | **p* values** |
| Troponin type | cTnI | 6 | 0.96 (0.37 to 2.51) | 95.6 | **<0.001** |
|  | cTnT | 1 | 3.16 (1.47 to 6.79) | 0.0 | - |
| Study design | PCS | 2 | 1.36 (0.89 to 2.07) | 28.0 | 0.239 |
|  | RCS | 5 | 1.04 (0.30 to 3.68) | 96.5 | **<0.001** |
| Troponin cutoff value | 0.1 to 0.5 µg/l | 1 | 1.81 (0.97 to 3.41) | 0.0 | - |
|  | 0.01 to 0.05 µg/l | 6 | 1.05 (0.39 to 2.87) | 95.8 | **<0.001** |
| Troponin assessment timepoint | Within 24 hrs | 3 | 1.76 (1.01 to 3.06) | 61.5 | **0.074** |
|  | Within 48 hrs | 1 | 3.05 (1.17 to 7.91) | 0.0 | - |
|  | Within 72 hrs | 1 | 1.15 (0.76 to 1.74) | 0.0 | - |
|  | Within 7 days | 2 | 0.35 (0.04 to 3.40) | 98.2 | **<0.001** |
| Estimation Method | ELISA | 1 | 3.16 (1.47 to 6.79) | 0.0 | - |
|  | CIA | 2 | 0.36 (0.04 to 3.53) | 98.4 | **<0.001** |
|  | RIA | 1 | 1.18 (0.77 to 1.80) | 0.0 | - |
|  | NR | 3 | 1.63 (0.96 to 2.77) | 50.6 | 0.132 |
| **Overall** | | | 1.13 (0.46 to 2.79) | 95.3 | **<0.001** |

***** **Bold values represent statistically significant heterogeneity (p-value < 0.05)**

**Table-S2 (A):** Summary of estimates based on subgroup analysis of last follow-up mortality in AIS.

| **Variables** | **Subgroups items** | **No. of studies** | **Risk Ratio**  **(95% Confidence Interval)** | **Degree of Heterogeneity** | |
| --- | --- | --- | --- | --- | --- |
|  |  |  |  | **I^2^ (%)** | **P values** |
| Troponin type | cTn | 2 | 1.89 (1.62 to 2.21) | 0.0 | 0.855 |
|  | cTnT | 1 | 3.93 (2.21 to 7.01) | 0.0 | - |
|  | cTnI | 10 | 2.86 (1.63 to 5.01) | 80.4 | **<0.001** |
|  | hs-cTnT | 7 | 6.02 (2.60 to 13.93) | 0.0 | 0.863 |
|  | hs-cTnI | 2 | 1.64 (1.15 to 2.35) | 0.0 | - |
| Study design | PCS | 14 | 2.34 (1.77 to 3.09) | 76.4 | **<0.001** |
|  | RCS | 8 | 2.57 (1.87 to 3.52) | 79.4 | **<0.001** |
| Troponin cutoff value | 0.1 to 0.5 µg/l | 3 | 1.71 (1.10 to 2.65) | 98.5 | **<0.001** |
|  | 0.01 to 0.05 µg/l | 19 | 2.58 (2.07 to 3.22) | 75.0 | **<0.001** |
| Troponin assessment timepoint | Within 24 hrs | 11 | 2.05 (1.60 to 2.63) | 77.6 | **<0.001** |
|  | Within 48 hrs | 6 | 2.90 (1.82 to 4.62) | 41.8 | 0.126 |
|  | Within 72 hrs | 1 | 1.80 (1.18 to 2.74) | 0.0 | - |
|  | Within 7 days | 4 | 3.48 (2.05 to 2.63) | 77.6 | **<0.001** |
| Estimation Method | ELISA | 4 | 1.75 (0.99 to 3.08) | 51.2 | **<0.001** |
|  | CIA | 12 | 2.36 (1.82 to 3.04) | 93.5 | **<0.001** |
|  | NR | 6 | 3.33 (1.95 to 5.66) | 76.3 | **<0.001** |
| **Overall** | | | 2.41 (1.98 to 2.93) | 76.5 | **<0.001** |

***** **Bold values represent statistically significant heterogeneity (p-value < 0.05)**

**Table-S2 (B):** Summary of estimates based on subgroup analysis of last follow-up mortality in SAH.

| **Variables** | **Subgroups items** | **No. of studies** | **Risk Ratio**  **(95% Confidence Interval)** | **Degree of Heterogeneity** | |
| --- | --- | --- | --- | --- | --- |
|  |  |  |  | **I^2^ (%)** | **p* values** |
| Troponin type | cTnI | 3 | 2.93 (1.93 to 4.46) | 68.7 | **0.041** |
|  | cTnT | 2 | 3.89 (2.08 to 7.28) | 0.0 | 0.567 |
|  | hs-cTnT | 1 | 2.72 (1.34 to 5.51) | 0.0 | - |
| Study design | PCS | 5 | 2.99 (2.09 to 4.27) | 43.2 | 0.134 |
|  | RCS | 1 | 3.51 (1.86 to 6.61) | 0.0 | - |
| Troponin cutoff value | 0.1 to 0.5 µg/L | 2 | 3.10 (1.94 to 4.96) | 0.0 | 0.614 |
|  | 0.01 to 0.05 µg/L | 4 | 3.07 (2.02 to 4.66) | 57.4 | 0.070 |
| Troponin assessment timepoint | Within 24 hrs | 2 | 2.13 (1.30 to 3.50) | 0.0 | 0.516 |
|  | Within 48 hrs | 1 | 3.51 (1.86 to 6.61) | 0.0 | - |
|  | Within 72 hrs | 3 | 4.01 (2.38 to 6.76) | 0.0 | - |
| Estimation Method | ELISA | 2 | 3.09 (1.59 to 6.01) | 0.0 | 0.434 |
|  | NR | 4 | 3.08 (2.17 to 4.37) | 55.3 | 0.082 |
| Overall | | | 3.08 (2.25 to 4.21) | 31.7 | 0.198 |

***** **Bold values represent statistically significant heterogeneity (p-value < 0.05)**
