# Supplementary material for: Elevated troponin levels as a predictor of mortality in patients with acute stroke: a systematic review and meta-analysis
Source: Front Neurol. 2024 Mar 25;15:1351925. doi: 10.3389/fneur.2024.1351925 (PMC10999611; doi:10.3389/fneur.2024.1351925)

Supplementary figure legends

Figure S1 (A-C): Funnel plot for the prediction of In-hospital mortality in (A) AIS (B) SAH and (C) ICH with respect to elevated cTn Levels

Figure S2 (A-B): Funnel plot for the prediction of Last follow-up mortality in (a) AIS and (B) SAH with respect to elevated cTn Levels

Figure S3 (A-C): Sensitivity plot for the prediction of In-hospital mortality in (A) AIS (B) SAH and (C) ICH with respect to elevated cTn Levels

Figure S4 (A-B): Sensitivity plot for the prediction of last follow-up mortality in (A) AIS and (B) SAH with respect to elevated cTn Levels

Figure S1 (A-C): Funnel plot for the prediction of In-hospital mortality in acute stroke with respect to elevated cTn Levels

(A)AIS: p-value= 0.45

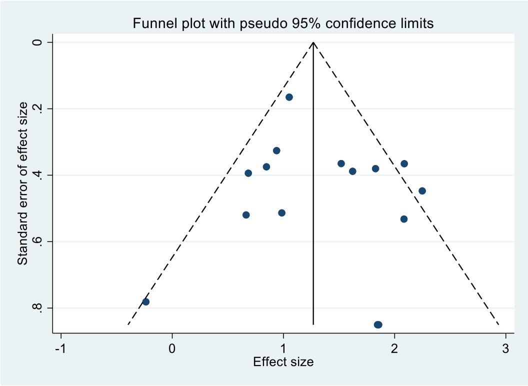

(B) SAH: p-value= 0.10

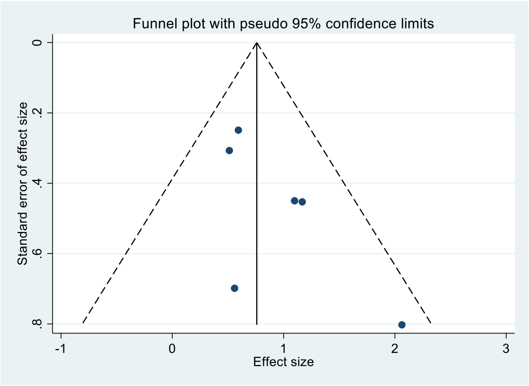

(C)ICH: p-value= 0.19

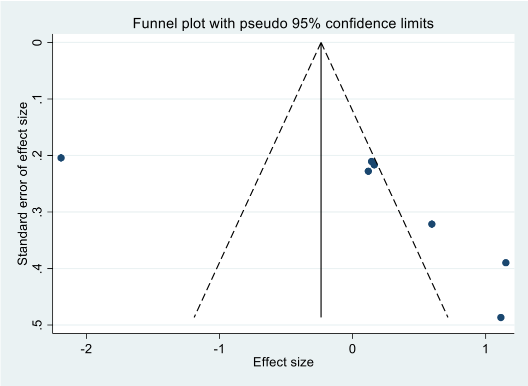

Figure S2 (A-B): Funnel plot for the prediction of Last follow-up mortality in acute stroke with respect to elevated cTn Levels

(A) AIS: p-value= 0.13

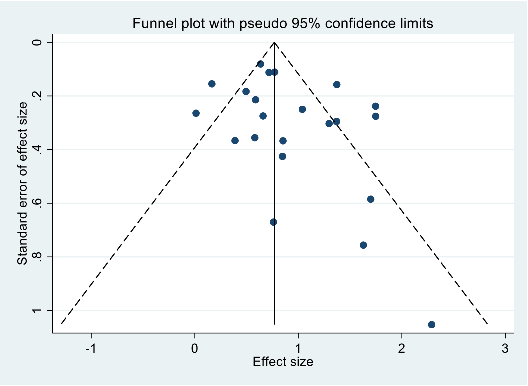

(B) SAH: p-value= 0.42

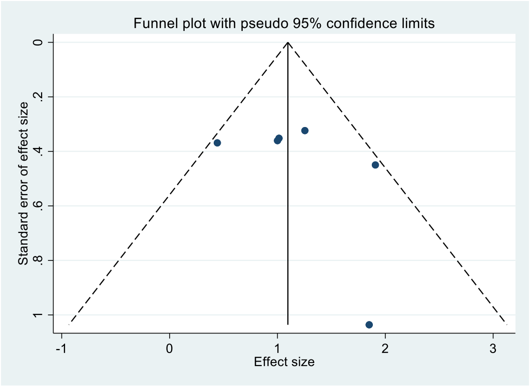

Figure S3(A): Sensitivity plot for the prediction of In-hospital mortality in AIS with respect to elevated cTn Levels

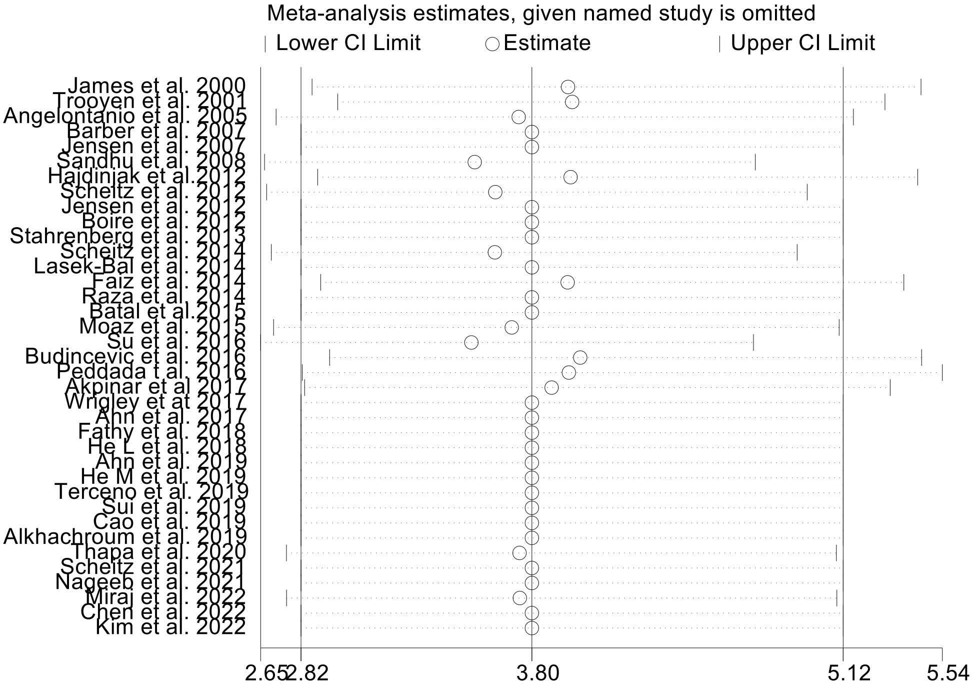

Figure S3(B): Sensitivity plot for the prediction of In-hospital mortality in SAH with respect to elevated cTn Levels

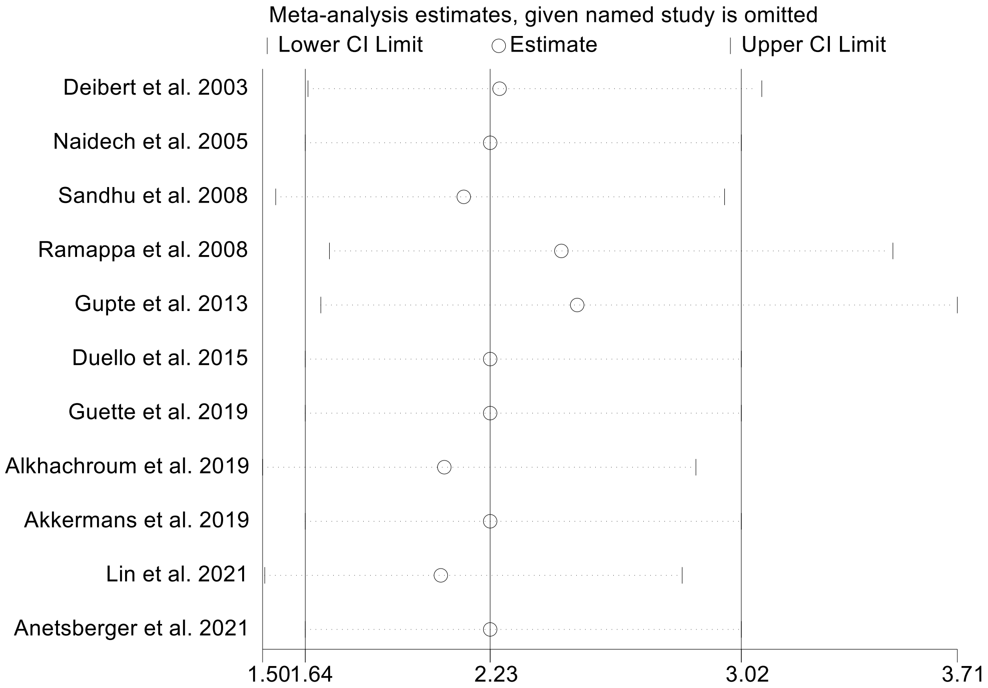

Figure S3(C): Sensitivity plot for the prediction of In hospital mortality in ICH with respect to elevated cTn Levels

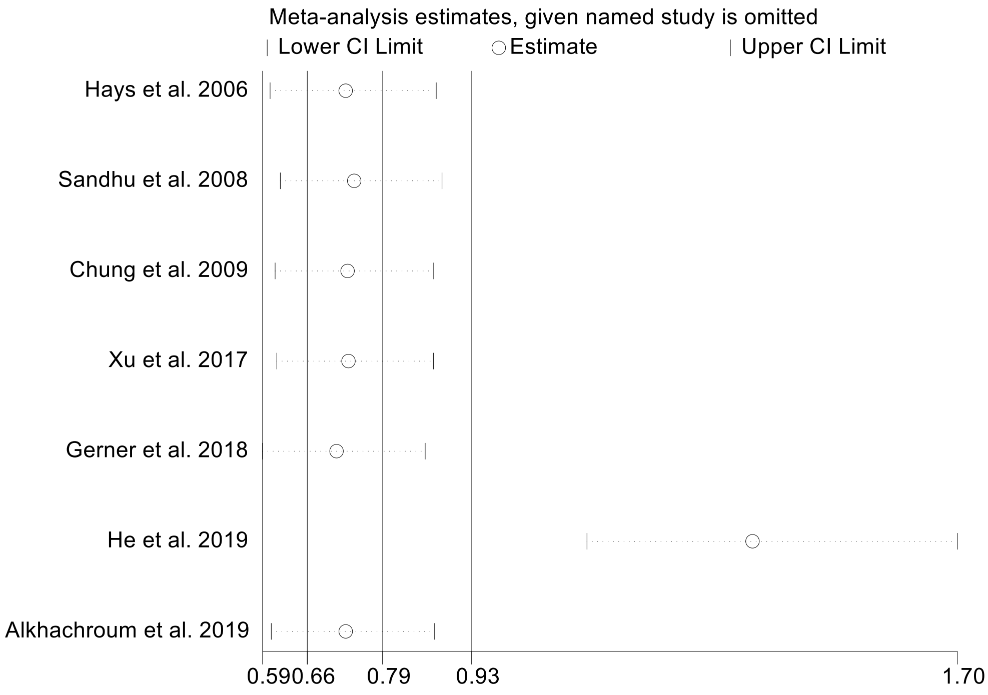

Figure S4(A): Sensitivity plot for the prediction of last follow-up mortality in AIS with respect to elevated cTn Levels

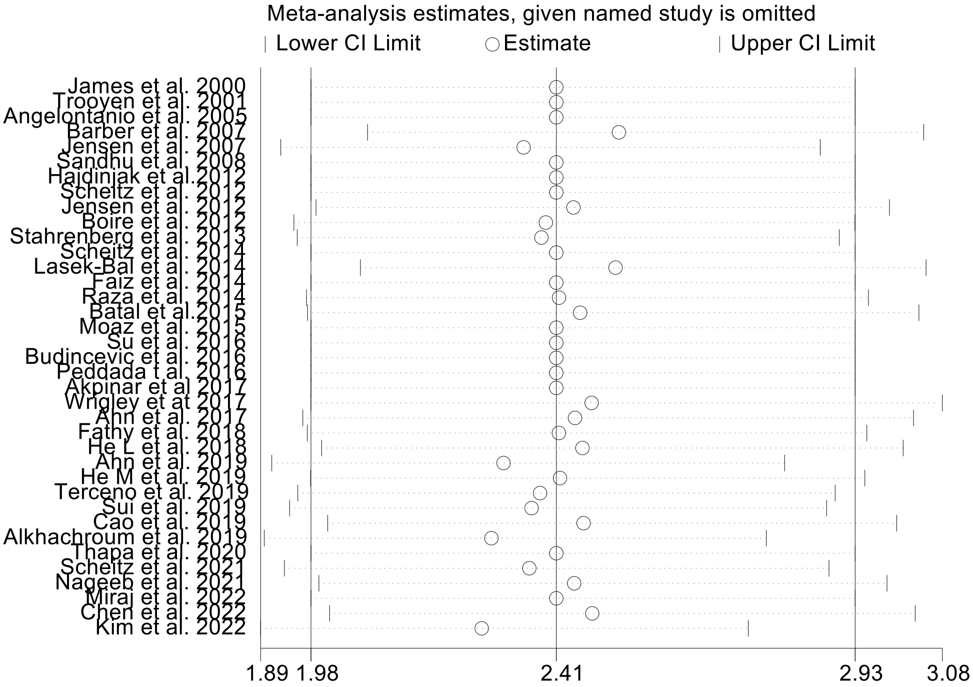

Figure S4(B): Sensitivity plot for the prediction of last follow-up mortality in SAH with respect to elevated cTn Levels

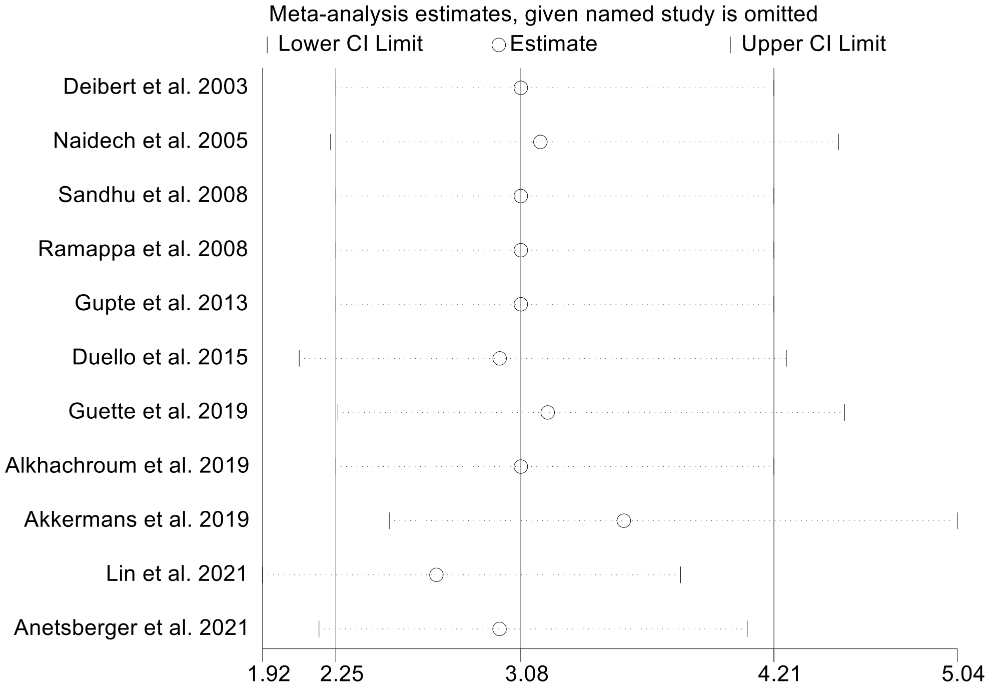

Supplement: Supplementary file 2 [file Image_1.pdf]
